# Pooled Testing for Effective Estimation of the Prevalence of *Schistosoma mansoni*

**DOI:** 10.4269/ajtmh.2012.12-0216

**Published:** 2012-11-07

**Authors:** Shira Mitchell, Marcello Pagano

**Affiliations:** Harvard University–Biostatistics, Boston, Massachusetts

## Abstract

Rapid and accurate identification of the prevalence of schistosomiasis is key for control and eradication of this devastating disease. The current screening standard for intestinal schistosomiasis is the Katz-Kato method, which look for eggs on slides of fecal matter. Although work has been done to estimate prevalence using the number of eggs on a slide, the procedure is much faster if the laboratory only reports the presence or absence of eggs on each slide. To further help reduce screening costs while maintaining accuracy, we propose a *pooled* method for estimating prevalence. We compare it to the standard *individualed* method, investigating differences in efficiency, measured by the number of slides read, and accuracy, measured by mean square error of estimation. Complication is introduced by the unknown and varying sensitivity of the procedure with population prevalence. The DeVlas model for the worm and egg distributions in the population describes how test sensitivity increases with age of the epidemic, as prevalence and intensity of infection increase, making the problem fundamentally different from earlier work in pooling. Previous literature discusses varying sensitivity with the number of positive samples within a pool, known as the “dilution effect.” We model both the dilution effect and varying sensitivity with population prevalence. For model parameter values suited to younger age groups, the *pooled* method has less than half the mean square error of the *individualed* method. Thus, we can use half as many slides while maintaining accuracy. Such savings might encourage more frequent measurements in regions where schistosomiasis is a serious but neglected problem.

## Introduction

Schistosomiasis (also known as Bilharzia) is a chronic disease caused by parasitic worms. Its intestinal form is responsible for severe liver and intestinal damage, physical growth retardation, and cognition and memory problems. The World Health Organization (WHO) reports that more than 200 million people are infected worldwide and an estimated 700 million people are at risk of infection because of their residence in tropical and subtropical areas, and in poor communities without access to safe drinking water and adequate sanitation. Young children are especially vulnerable to infection because of their hygiene and play habits, and the symptoms are quite harmful to them, impairing learning ability and physical development, and even sometimes causing death.

The current who strategy for schistosomiasis control focuses on reducing disease through periodic, targeted treatment with praziquantel. The WHO guidelines identify three strategies based on community prevalence of infection: 1) in communities with a high prevalence (more than 50% infected, we call the *high* group) universal treatment is conducted once a year; 2) in communities with a moderate prevalence (more than 20% infected but < 50%, we call the *moderate* group) school-age children are treated once every 2 years; and 3) in communities with a low prevalence (< 20% infected, we call the *low* group) chemotherapy should be available in health facilities for treatment of suspected cases.^1^ To implement the WHO strategy, we must effectively classify each community into one of these three categories. A strategy for achieving this aim is the subject of this work.

True *prevalence* of active infection is defined as the proportion of individuals with at least one worm-pair (one male and one female, otherwise if only one gender is present, they cannot reproduce). Most control programs are based on the detection of eggs by fecal smears on 47 mg slides using the Kato-Katz technique. The *observed prevalence* is defined as the proportion of individuals who show at least one positive egg count.^2^ In contrast, the observed prevalence is dependent on the quantity of stool examined in the sample, the number of samples collected (and at which time intervals), and the average worm load (indirectly measured by *intensity*—eggs/g feces). This is caused by the possibility that a person has worms but that eggs are not present in the stool on a given day, or the eggs are present in the stool, but not in the smear taken from the sample and placed on the slide, or the eggs are on the slide but not seen by the reader. Thus, one has to be careful when designing policy based on observed prevalence. To be concrete, and motivated by the interest in the propagation of the infection, we focus on prevalence as the percentage of individuals with at least one worm-pair. In this way, we can compare prevalence estimates using different algorithms by adjusting the observed prevalence appropriately. Population-level treatment and control are dependent on the measurement of prevalence, thus it is important to investigate the ways of making the measurement as accurately as one can within a reasonable budget, and that is the goal of this work.

Current WHO recommendations for estimating prevalence are based on sample surveys of 50 children/school within the defined ecological zones^1^; this sample size was possibly selected because it was considered to be the number of sample slides that a survey team examines in a single day. Such an approach typically involves a survey team of several staff moving with a single vehicle, and necessitates entry and analysis of survey data. It is therefore often considered prohibitively expensive for a national program to sustain parasitological surveys on a large scale when this approach is used.^3^

To reduce screening costs by reducing the number of slides examined, but at the same time not sacrificing any accuracy, we propose a new way to measure prevalence of schistosomiasis that we call *pooled testing*. The current standard, against which we contrast our proposed method, we call *individualed*, and it examines 47 mg of feces per slide, taken as four snips from one child's fecal sample smeared on a slide. The pooled method combines snips, of about 12 mg each, from separate fecal samples from four children smeared on a single slide. Both techniques have an *observed* prevalence that will underestimate the true prevalence (based on the worm-pair definition). Thus, we need to adjust either estimate (the individualed or the pooled) upward to overcome the bias and come closer to the true prevalence.

Previous literature on pooling methods discusses the varying sensitivity of the pooled test with the number of positive samples within a pool. This is known as the “dilution effect.”^4^ Our model takes into account both the dilution effect and the varying sensitivity of the test with intensity of infection, as modeled by the DeVlas model introduced in the next section.

### The model.

The goal of this work is to estimate the true prevalence, the proportion of individuals with at least one worm-pair. The data consist of positive (eggs found) or negative (eggs not found) readings of fecal smear slides, however the slides are constituted. We need to understand the relationship between the true prevalence and probability that eggs are found in the laboratory testing procedure (the sensitivity). Thus, it is necessary to model how the number of eggs per fecal sample varies from person to person and from day to day.

To this end, let *P*(*Y* = *y*; *h*_0_*x*,*r*) be the probability of finding *y* eggs in a stool smear (∼12 mg of fecal matter) from a person with *x* worm-pairs. Let *h*_0_ be the number of eggs per smear per worm-pair. Note that the distribution of *Y* incorporates the variability in egg output in the stool, the variability in the number of eggs captured in the smear, and the variability in what the laboratory technician can actually count. DeVlas considers the negative binomial model where *Y* ∼ NegBin(*h*_0_*x*,*r*) with mean *h*_0_*x* and index of aggregation *r,*^5^ because *r* (the index of aggregation in the distribution of egg counts) increases, the variance decreases. The parameter *r* can also account for imprecision in the measurement of 12 mg of fecal matter per smear. For example, if one smear is actually 16 mg, and another is 8 mg, the difference in egg counts from smear to smear is more variable, which can be built into the model with a smaller value for *r*. We see below in simulations that the relative benefit of pooling (compared with the individualed method) does not seem to be sensitive to the value of *r*.

If *n_m_* and *n_f_* represent the number of male and female worms, respectively, (*n* = *n_m_* + *n_f_*) then *x* = min(*n_m_*,*n_f_*) is the number of worm-pairs. DeVlas considers *n_m_* and *n_f_* ∼ Bin(*n*,1/2). Let *P*(*X* = *x*∣*N* = *n*) be the probability of having *x* worm-pairs for an individual with worm load *n*. Let *P*(*N* = *n*; *M*,*k*) be the probability of having *n* worms. Let *N* ∼ NegBin(*M*,*k*) have mean *M* and index of aggregation *k*. As *k* (the index of aggregation in the distribution of worms in the population) increases, the variance decreases. Small values of *k* indicate more aggregation and relative overdispersion, with the worm counts highly concentrated in a small section of the population. This situation arises in populations with a low level of immunity, where variation in exposure is not countered by the development of immunity. Such low levels of immunity are seen in younger age groups, where there are lower values of *k*^5^; another reason there could be a high level of overdispersion in worm load (i.e., a low value for *k*) would be community variation in exposure to infection. Such heterogeneity could arise from a community being composed of a variety of occupations.^5^ Thus, when modeling the value of *k* in a community; one should consider age and homogeneity of exposure. If one chooses to perform Bayesian inference (rather than maximum likelihood), one might then have a prior distribution on *k* and the prevalence *p*, which together can be used to compute a prior distribution for *M* (because prevalence *p* is a function of *k* and *M*). We explore this Bayesian formulation further in Reference 6. The overall distribution of the number of eggs per smear (*y*) is *P*(*Y* = *y*; *M*,*k*,*h*_0_,*r*) (see the Appendix in Reference 5).

Let *c* be the number of smears per slide. In the literature, *c* is referred to as the *composite sample size*, or the *pool size*. We focus on the case of *c* = 4 because of common field practice, but our derivations are kept general and our simulation program can accommodate other values of *c*. To describe collecting *c* = 4 smears from 1 person on the same day we refer to *h*_0_ as the number of eggs per smear per worm-pair, so *h* = *ch*_0_ is the number of eggs per slide (i.e., sample) per worm-pair. We define prevalence as the probability of having at least one worm-pair, so *p* = *P*[*X* > 0] (see Reference 7, Box 1.) We define the *c*-smear sensitivity as the sensitivity when using *c* smears from the same individual. See Appendix for mathematical details to derive an expression for *sens_c_*, the *c*-smear sensitivity.

## Individualed Prevalence Estimator

In this section we discuss the estimator of prevalence using the individualed method. Let *P*_1_ be the probability of a positive slide, which is a function of the prevalence *p*. Let *m* be the number of slides tested and *W* the number of positive slides, with 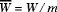. Letting *sens*_4_ = P_r_[*Y*_1_ + *Y*_2_ + *Y*_3_ + *Y*_4_ > 0∣*X* > 0] be the sensitivity of the test using the individualed method with four smears from one person, we see that *P*_1_(*p*) is

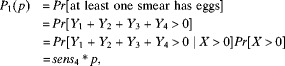
because we assume no “eggs” will be spotted in a smear coming from a person with no worm-pairs, and thus take the specificity of the measurements to be one—i.e.,

*Pr*[find no eggs in smear∣person has no worm-pairs] = *P*[*Y* = 0∣*X* = 0] is 1. Solving for *p* we have the mle, 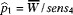, which is unbiased, if we know *sens*_4_. The variance is

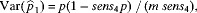
if the *sens*_4_ used in the estimator is the true sensitivity. Note that 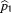 might be greater than one. Therefore, define a truncated estimator,

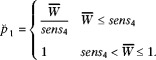


For values of the parameters we are investigating, 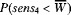 is zero up to at least two significant digits, and furthermore, it will be obvious we are in this unlikely case if our prevalence estimate is greater than 1, so we focus on the non-truncated 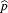 henceforth.

## Pooled Prevalence Estimator

Now consider the estimator of prevalence using the pooled method. Define *W* as before, with the probability of a positive slide in the pooled case (the subscript “*c*” denotes that the slide consists of smears from *c* separate individuals). Again, we maximize the likelihood to get 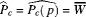. Letting *sens*_1_ = *P*[*Y* > 0∣*X* > 0] = *P*[*Y* > 0] / *P*[*X* > 0] (the sensitivity for a single smear), and recalling that the specificity is one,

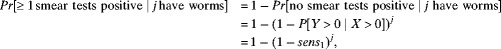
and

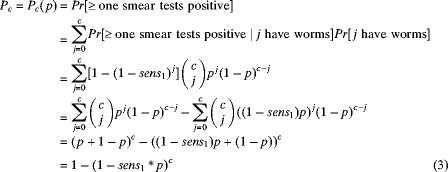


Solving for *p* we have the mle, 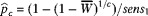, which is biased (see Bias and bias correction section and Reference 8). The truncated estimator is

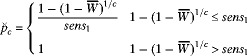


Although it is possible that 1 − (1 − *W*)^1/*c*^ > *sens*_1_, for likely values of model parameters, it is a very unlikely event. It will be obvious we are in this unlikely case if our prevalence estimate is greater than 1. Thus, we focus on the estimator 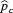.

### Bias and bias correction.

Recall that *c* is the number of smears per slide. In the pooled method we propose, we can consider a slide to be a pool, as in the literature (see References 8–11 for more about pooled testing). Note that our setting differs from the settings in the literature, which either uses a prefect test or a test whose sensitivity does not get diluted in a pool, which is a more realistic model in our setting of testing for estimation of schistosomiasis prevalence.

Let *P_c_* be the probability of a positive slide in the pooled case. With a perfect test, a slide is negative if and only if all *c* individuals are not infected, therefore 1 − *P_c_* = (1 − *p*)^*c*^, or *p* = 1 − (1 − *P_c_*)^1/*c*^. The quantity *H*(*P*_*c*_) = 1 − (1 − *P*_*c*_)^1/*c*^ is called the *prevalence transformation*.^8^ It takes the probability of a positive pool and transforms it to the probability of a positive individual. Because we are assuming a test with imperfect sensitivity (i.e., *sens*_1_ < 1), we denote the prevalence transformation in our case (with *P_c_* given by Eq. [3]) by 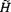. For *c* > 1, *H*(*t*)(and 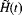) is monotone increasing in *t*, so we can reduce the bias in 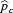 by shrinking *P_c_* toward zero before applying the prevalence transformation.^8^

The *shrinking estimator* is 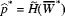 where 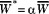 for some α, 0 ≤ α ≤ 1. A natural choice is α, 0 ≤ α ≤ 1. A natural choice is α = 1 − *b* / (*n* + *d*), for some positive numbers *b*, *d* which act as adjustable parameters to regulate the degree of shrinking. For the *Burrows' estimator* 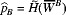 where 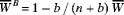 therefore α = 1 − *b* / (*n* + *b*), in other words, it is the shrinking estimator where we choose *b* = *d*. We choose *b* to eliminate the order *n*^− 1^ term in the bias (substituting in 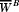 into the above Taylor expansion of 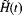 to evaluate the 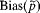, and get *b* = (*c* − 1)/(2*c*), see Reference 8 for details.
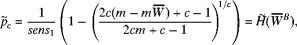
compare with 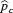 in Eq. (5), our more biased estimator:

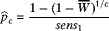


**Figure 1.**
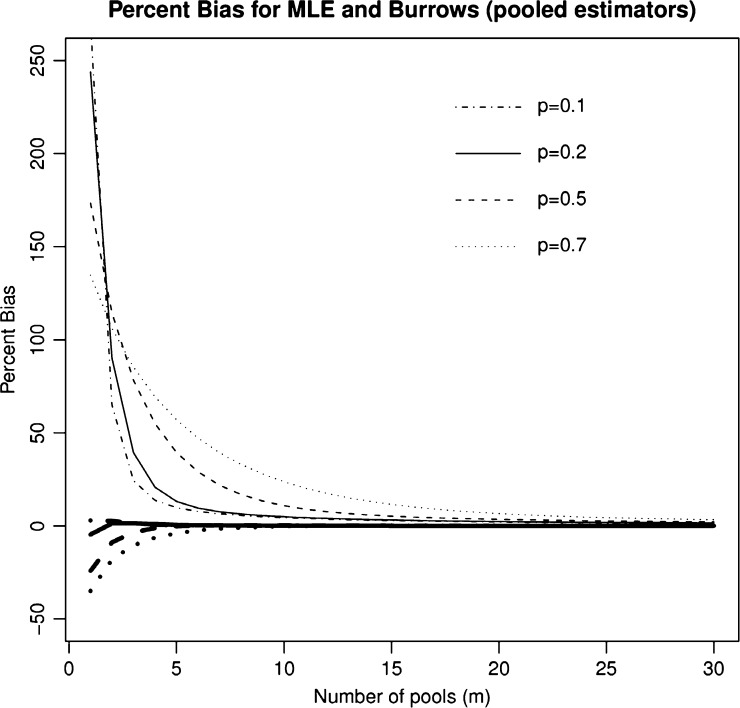
Percent Bias in the mle (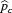, thin lines) and Burrows (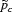, bold lines) estimators for pool size *c* = 4 as a function of number of slides (*m*) for low, moderate, and high prevalence settings: Using the Burrows estimator results in a reduction in finite sample bias. Note that beyond *m* = 15 slides the bias is nearly zero.

We plot the percent bias in the prevalence estimator for 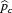 and 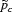 for *c* = 4 in Figure 1. The bias of the mle is

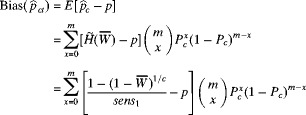
*if* the *sens*_1_ used in the estimator is the true sensitivity. The bias of the Burrows estimator is

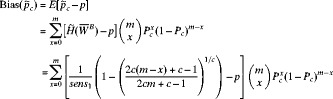
(again, if *sens*_1_ is the true sensitivity.) The percent bias is 100*Bias(estimator)/*p*. We choose to use the Burrows correction for the pooled estimator because it significantly reduces the bias in the pooled estimator. Thus, when dealing with the case of taking more than 15 slides per school, expect the bias of the Burrows-corrected estimator to be nearly zero.

#### Key point.

The maximum likelihood estimator (mle) overestimates the prevalence; therefore, we use the Burrows estimator (a type of so-called “shrinking estimator”) that shrinks the estimate slightly, to reduce the bias of our estimator.

### Asymptotic mse ratio of the estimators.

Because one component of the screening costs for schistosomiasis is dominated by the number of slides examined, we wish to know which technique gives a more accurate estimate of prevalence (and risk category) given the same number of slides. To evaluate the effect of pooling we examine the ratio of the asymptotic mean square error (mse) for the pooled estimator 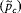 to the asymptotic mse for the individualed estimator 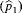. Again, in this section we assume that the *sens_c_* and *sens*_1_ used in the estimators are the true sensitivities. From the Individualed Prevalence Estimator section, we know that 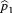 is unbiased with the computed variance. Thus, we get the mses from the variance for the individualed estimator Eq. (2), which is unbiased, and

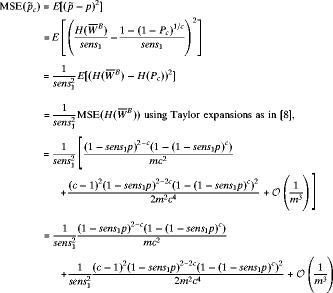
Then, the asymptotic ratio (as *m* → ∞) is

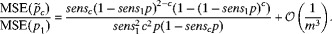


**Figure 2.**
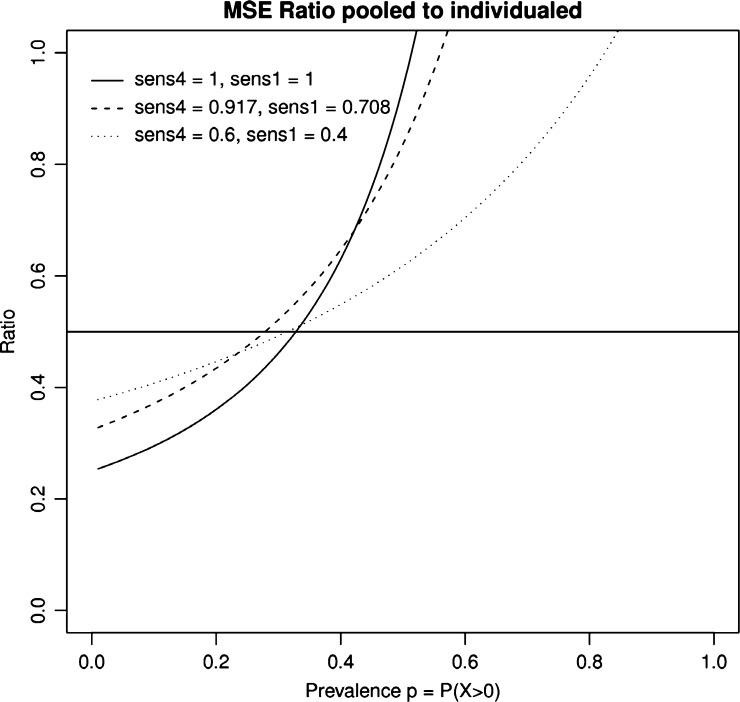
MSE ratio pooled to individualed: For three example settings of the *sens*_4_ and *sens*_1_.

We plot this asymptotic mse ratio, as a function of the prevalence *p*, for *c* = 4, and for three different values for the sensitivities, *sens*_4_ and *sens*_1_ (see Figure 2). We choose values to show the case of a perfect test, values from the literature,^13^ which found that “the overall sensitivity of a single Kato-Katz smear was 70.8%, and it increased with each additional slide to reach 91.7% on examining four smears,” and finally, values to indicate a lower range of sensitivity, because our technique uses less fecal matter than^13^ to fit all smears onto a single slide. Of importance, the sensitivities from Reference 13 were based on a single stool sample, so the relative change in sensitivity when increasing to four times the fecal matter can be expected to be similar. Increasing the number of stool samples across time has been found to increase the sensitivity more drastically.^14^ Figure 2 shows that the mse of the pooled estimator is roughly half that of the individualed estimator at prevalences below roughly 30%. This is of interest because we can read half as many slides to achieve the same accuracy of prevalence estimation. Note that we are in some sense making an unfair comparison because the slides from the pooling technique use fecal matter from more individuals. However, the comparison is the comparison of interest because the cost is dominated by the cost of slides and the cost of reading each slide not on the amount of fecal matter used.

Note also that with a perfect test, below prevalences of roughly 50%, the pooled estimator has smaller asymptotic mse than the individualed estimator. The poor performance of a pooled estimator at high prevalences has been well studied (see References 8–11) and results from the fact that getting all positive pools is not informative. Note however, this upper bound gets higher as the sensitivities decrease, because lower sensitivity lowers the probability of all positive pools.

**Figure 3.**
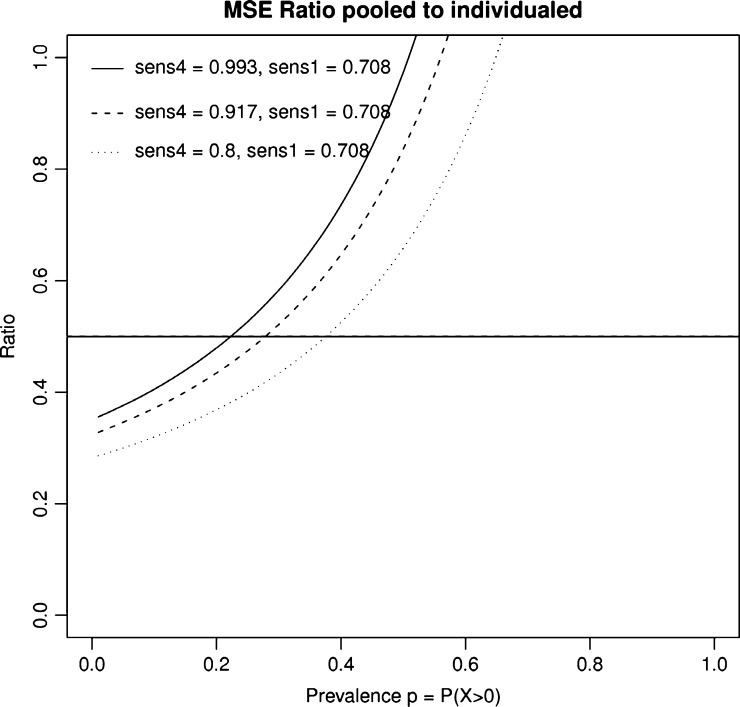
MSE ratio pooled to individualed: Comparing *sens*_4_ < 1 − (1 − *sens*_1_)^4^ to *sens*_4_ = 1 − (1 − *sens*_1_)^4^.

Intuitively, the four smears from the same individual may be redundant, because egg counts per smear are positively correlated.† †If one smear has eggs, then the individual is clearly infected (i.e., has worms), and the worms are producing eggs on that day, so the stool contains eggs. Thus, the other three smears are more likely to have eggs and therefore to provide redundant information. The DeVlas model described previously accounts for this because of the correlation of egg counts within an infected individual. Thus, in general, *sens*_4_ < 1 − (1 − *sens*_1_)^4^. This holds for the observed sensitivities in Reference 13 where 0.917 < 1 − (1 − 0.708)^4^ = 0.993 and more extremely for 0.6 < 1 − (1 − 0.4)^4^ = 0.8704, our third example of sensitivities. We see in Figure 3 that this property of the sensitivities pushes the curve downward, making pooling more efficient relative to not pooling. Furthermore, as *sens*_1_ gets closer and closer to *sens*_4_, we get closer and closer to the usual pooling setting in which it is assumed that sensitivity does not decrease with pooling. In other words, having only one infected smear on a slide is just as easily detected as having four infected smears. This is likely not true in general for schistosomiasis, but may be close to true in situations of a high intensity of infection, when infected people have many worms, which therefore produce many eggs. This is likely to hold true in regions where there has been no treatment available for a long time, so infections have been allowed to grow stronger and worms have multiplied enormously. Thus, assuming constant and known values for *sens*_1_ and *sens*_4_ for the population, we see from the previous derivation that pooling is much more efficient, allowing for reading half the slides to achieve the same level of accuracy.

**Figure 4.**
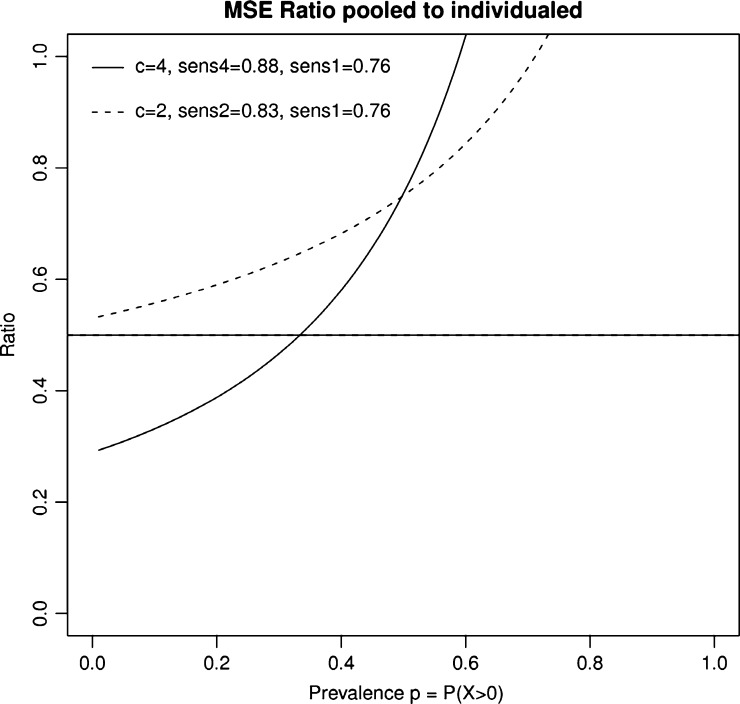
MSE ratio pooled to individualed: Comparing *c* = 4 to *c* = 2 for *sens*_4_ = 0.88, *sens*_2_ = 0.83, and *sens*_1_ = 0.76.

In Figure 4, we examine the effect of decreasing to a pool size of *c* = 2. We obtain values for *sens*_1_, *sens*_2_, *sens*_4_ by the DeVlas model with parameters *M* = 500, *k* = 0.2, *h*_0_ = 0.085, *r* = 1.6, and plot the ratio of pooled/individualed mse for a pool size of two and four. We see that a pool size of two is less beneficial for lower prevalences, but does slightly better at higher prevalences (above roughly 50%). Because we are most likely dealing with prevalences below 50% (and those above 50% are all treated the same according to the WHO recommendations), we decided to proceed with the pool size of four. Beyond four is likely infeasible as a laboratory procedure.

Figure 2 assumes constant values for *sens*_4_ and *sens*_1_, which are *known* and chosen correctly in the estimators. In reality however, they depend on the parameters *M*,*k*,*h*_0_,*r*. Because we do not know the parameter values, we do not know the true values of *sens*_4_ and *sens*_1_ or the true prevalence. Because prevalence is a function of *M* and *k*, the values for *sens*_4_ and *sens*_1_ will *change* with the prevalence. The asymptotic expression for mse above does not incorporate this added complexity. To get a picture of how the mse ratio behaves with a finite number of slides (we use the current standard of *m* = 50 slides) and to explore how the mse ratio is affected by varying the parameters *M*,*k*,*h*_0_,*r* in the model, we turn to simulations.

#### Key point.

The mean square error of an estimator measures the square of the average amount by which the estimator misses the quantity we are trying to estimate (for us, the prevalence). It plays the role of the variance when dealing with a biased estimator. By asymptotic, we mean we examine this error as the sample size approaches infinity. We compare the pooled and individualed estimators by their asymptotic mean square error to see which one does better at estimating prevalence as the sample size approaches infinity. Figure 2 shows us that the mean square error of the pooled estimator is roughly half that of the individualed estimator at prevalences below roughly 30%. This is of interest because we can read half as many slides to achieve the same accuracy of prevalence estimation.

## Simulations

For each plot, we fix three of the four parameters *M*,*k*,*h*_0_, and *r* and vary the fourth parameter to study the variance, bias, and mse change for the pooled and individualed estimators. In contrast to the asymptotic analysis, we now allow the true sensitivities to vary with the varying parameter values. In other words, for the individualed method, we pick a random number of worms for the individual (from the Negative Binomial in the DeVlas model above), and given this worm count pick an egg count for a quantity of 47 mg of stool. If this count is nonzero, we say we have a positive test result. Thus, in our simulation we allow each individual in a population to have their own sensitivity, which is a function of their own worm count, in other words: *sens*(*x*) = *P*[*Y* > 0 ∣ *X* = *x*]. Similarly, in the pooled method, we pick four random numbers of worms and for each of these worm counts we pick egg counts for a quantity of 12 mg of stool.

Note, of course, that the estimators of prevalence must have fixed values for *sens*_4_ and *sens*_1_, so we estimate them based on parameter values in the center of the ranges over which they vary. Thus, we expect the bias to be near zero in a neighborhood of these values (where we “guess” the sensitivities correctly) and to get larger in absolute value as the parameter values deviate further from these center values.

We ran 5,000 simulations for each value of the four parameters. We chose parameter values to be representative of populations of young children in sub-Saharan Africa, and prevalences between 0 and slightly above 50%, because prevalences well above 50% are easily identified. At *k* = 0.2 and *M* = 20, the prevalence is ∼50%, the boundary between the moderate and high groups. We see that in general we might expect lower values of *k*, the aggregation parameter, among younger children, so we focus on such lower values of *k* (see Reference 5). We fix *h* = 0.05 (for four smears, *h*_0_ = 0.05/4 for one smear), which reflects an egg per worm-pair output typical of Africa and the African diet (see Reference 5, p. 456). Finally, we choose *r* = 1.6 to reflect a moderate level of variability of egg counts for an individual.

**Figure 5.**
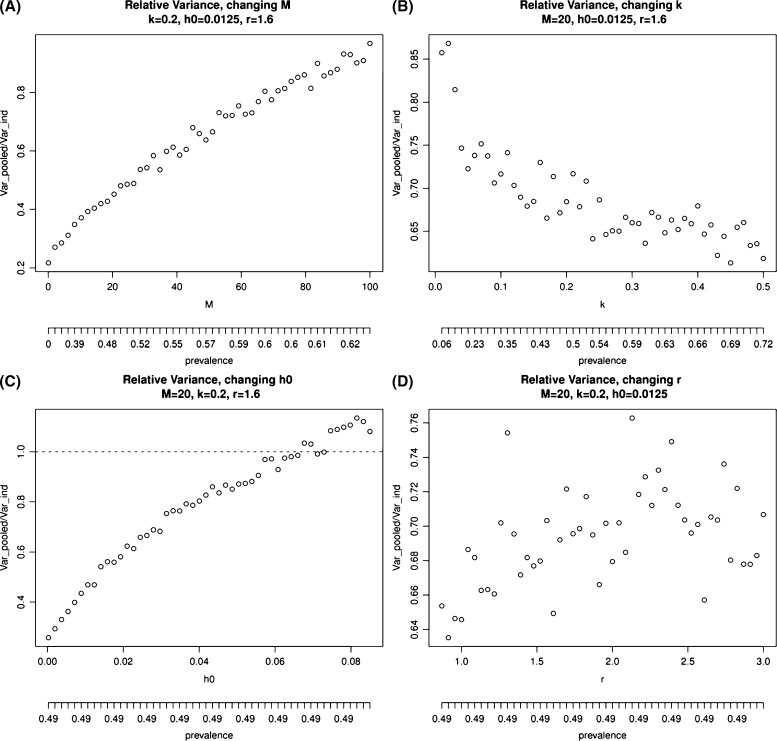
Relative Variance: (**A**) Relative Variance, changing *M*; (**B**) Relative Variance, changing *k*; (**C**) Relative Variance, changing *h;* (**D**) Relative Variance, changing *r*.

Comparing the variance of the pooled to the individualed estimator, in Figure 5B, we see that holding *M* = 20 fixed and changing *k* from 0 to 0.5, we have a relative variance (pooled to individualed) that drops to roughly 0.65.

Changing *h*_0_ and *r* does not change the prevalence, and in Figure 5C and D we look at a prevalence of 49% (near 50% cutoff). As *h*_0_, the egg output per worm-pair increases, the relative variance increases but remains well below 1. As *r*, the aggregation for egg output, increases (meaning that egg output variability decreases) the relative variance increases slightly, but the correlation does not appear very strong.

**Figure 6.**
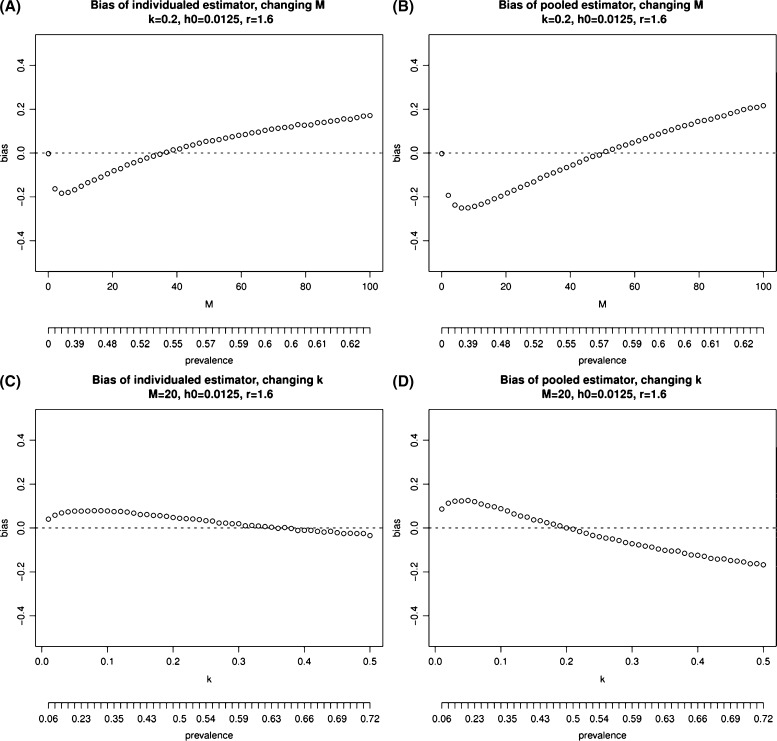
Comparing the Bias. (**A**) Bias for individualed, changing **M**; (**B**) Bias for pooled, changing **M**; (**C**) Bias for individualed, changing **k**; (**D**) Bias for pooled, changing **k**.

**Figure 7.**
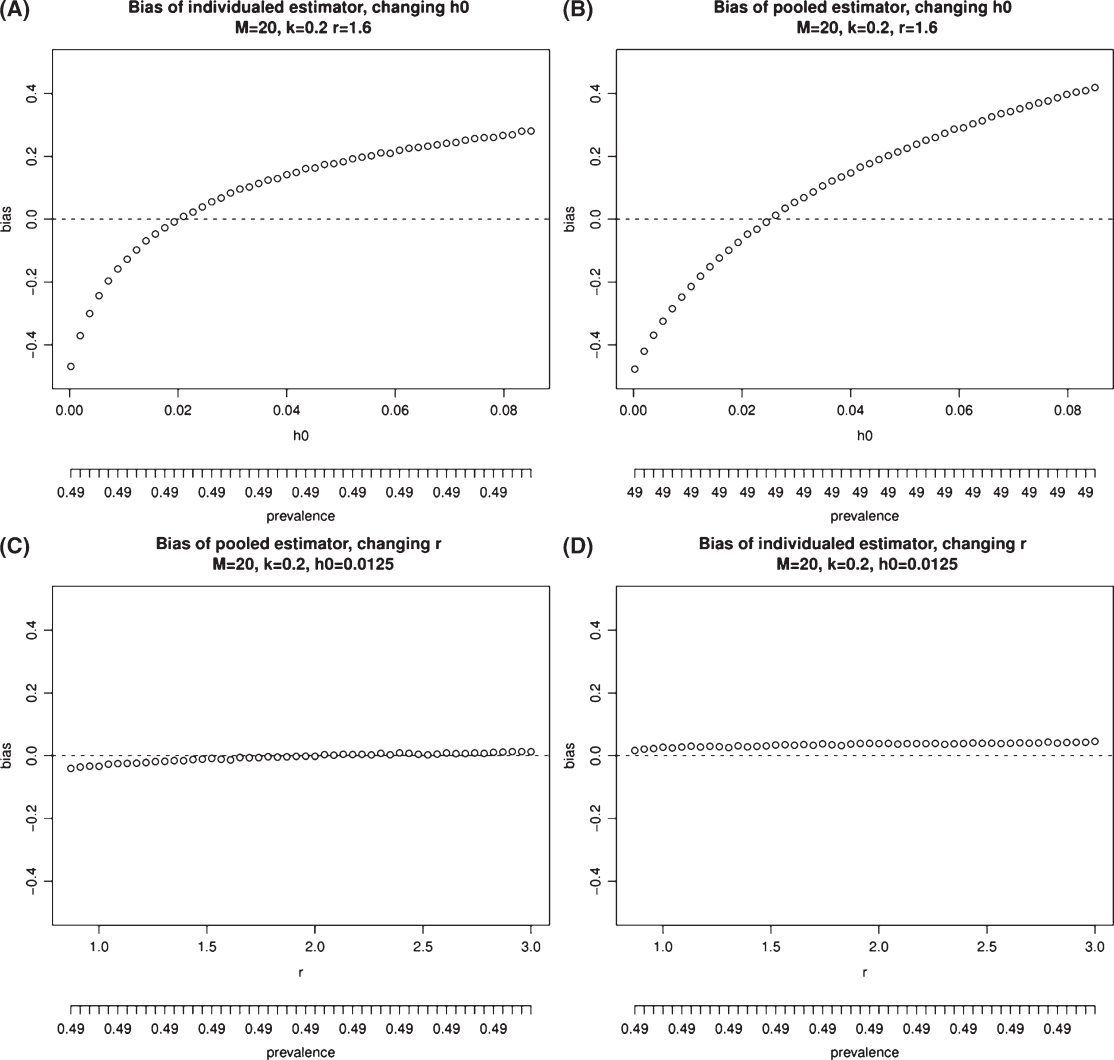
Comparing the Bias: (**A**) Bias for individualed, changing *h*; (**B**) Bias for pooled, changing *h*; (**C**) Bias for individualed, changing *r*; (**D**) Bias for pooled, changing *r*.

Figures 6 and 7 compare the bias of the individualed and the pooled estimators. Both have zero bias at the parameter values chosen to estimate the sensitivity (*sens*_4_ and *sens*_1_). We can see that the *sens*_1_ = *P*[*Y* > 0 ∣ *X* > *x*] = *P*[*Y* > 0]/*P*[*X* > 0] is more sensitive to choice of parameters (*M*,*k*,*h*_0_, and *r*) than *sens*_4_ = *Pr*[*Y*_1_ + *Y*_2_ + *Y*_3_ + *Y*_4_ > 0]/*Pr*[*X* > 0], therefore the bias will usually be worse in the pooling case if we do not know the values for *M*,*k*,*h*_0_,*r*.

**Figure 8.**
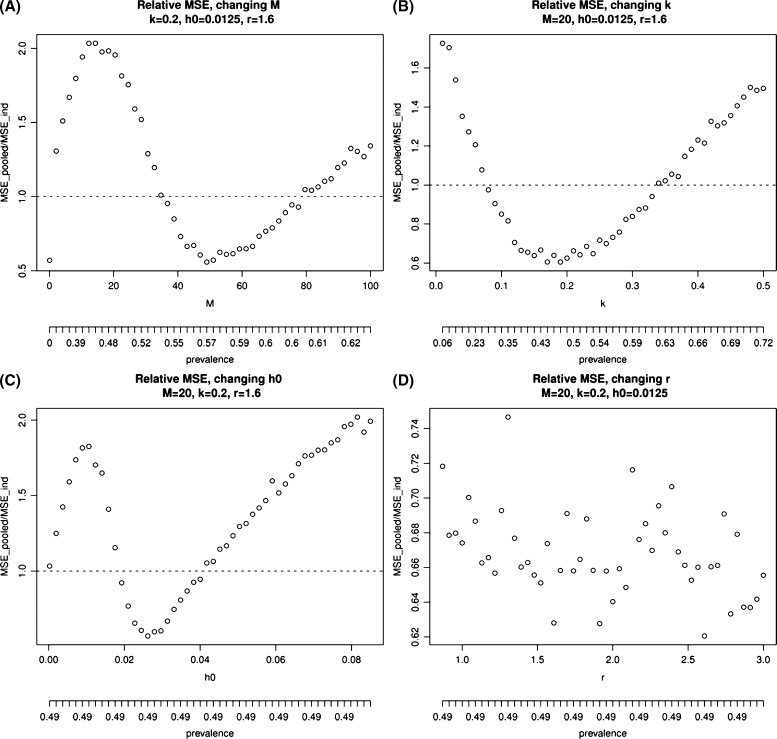
Relative mse: (**A**) Relative MSE, changing **M**; (**B**) Relative MSE, changing **k**; (**C**) Relative MSE, changing **h**; (**D**) Relative MSE, changing **r**.

Comparing the mse of the pooled and individualed estimators in Figure 8 we see that the relative mse is mostly below 1, even where the bias of the pooled estimator is higher than the individualed, because the variance of the pooled estimator is much lower than the variance of the individualed estimator. This argues for preferring the pooled estimator over the individualed estimator.

## Estimating Prevalence

We are trapped in circularity when trying to estimate prevalence, because we need to know the prevalence to know the sensitivity, which is required to estimate the prevalence unbiasedly. Below we suggest a method to arrive at an estimate of prevalence that takes into account the dependence of sensitivity on the prevalence by using an iterative technique.

We need values for *sens*_4_ or *sens*_1_ to report an unbiased individualed or pooled estimate, respectively. However, *sens*_4_ and *sens*_1_ are functions of the model parameters *M*,*k*,*h*_0_,*r*, and in particular, vary greatly with prevalence (a function of *M*,*k*). Thus, to estimate prevalence unbiasedly, we are required to already know the prevalence, an obvious circularity. In an attempt to combat this problem, we begin with an initial guess at the prevalence 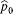 (say, the observed prevalence, i.e. ignoring the sensitivity issue). With some knowledge of our population (age group, etc.) we set *k*, the aggregation parameter, and using *p*_0_ get a value for *M*. On the basis of these *k*,*M* we compute the corresponding 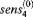 and 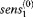 based on 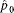. We can then use these sensitivities to get a new estimate of prevalence 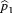 and again use this to compute corresponding 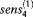 and 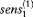. We can continue in this iterative manner until we converge to an estimate of prevalence. If we start close enough to the unbiased prevalence, this method will converge to the unbiased prevalence.

We can define 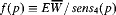, so *p*_*n*+1_ = *f*(*p*_*n*_). The prevalence is a fixed point of *f*. From numerical analysis, we know that *f* converges to the fixed point *p* (the true prevalence) if *f* is continuously differentiable in an open neighborhood of a fixed point *p* and ∣*f*′(*p*)∣ < 1 (see Reference 15, pp. 226–233). This property is usually difficult to prove, so we recommend performing a few iterations (which are very fast) and assessing convergence.

Similarly for the pooled case, where we define

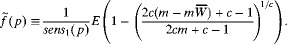
For large enough sample sizes the bias of the Burrows estimator is essentially zero, so we may assume we want the fixed point here as well to get the true prevalence. Again, to prove convergence, one must check that 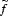 is continuously differentiable in an open neighborhood of a fixed point *p* and that 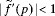.

We are not given the true 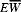 or 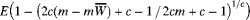 but these can be consistently estimated by 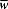 and 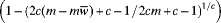 so we can expect convergence to 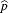 such that 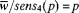 and 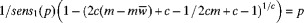, respectively. These iterations do not take long to converge and the result is an estimate of prevalence that takes into account the variance of sensitivity with prevalence.

## Simulations from Field Data

We used school-level data from schools in Uganda (296 schools, average prevalence of *Schistosoma mansoni* per school: 28%^16^), Tanzania (143 schools, average prevalence of *S. mansoni* per school: 4.4%^17^), Mali (454 schools, average prevalence of *S. mansoni* per school: 10%^18^), and Cameroon (402 schools, average prevalence of *S. mansoni* per school: 7.3%^19^).

Using data from these four countries we compiled a list of 1295 schools with the measured prevalences from the field. For the purpose of simulation, we took these prevalences to be truth (likely an underestimate, because of the poor sensitivity of the test). For each school we performed five methods: testing of 12, 25, and 50 individualed slides, and testing of 12 slides composed of four children each, and testing of 25 slides composed of four children each. We used 0.9 for the 4-smear sensitivity (*sens*_4_) and 0.7 for the single-smear sensitivity (*sens*_1_), as was found in Reference 13. We use a simplifying assumption that this sensitivity is constant and known. We preformed 5,000 simulations, and report the average results in Table 1. It provides evidence of the benefits of pooling.

We summarize the results in two summary measures (see Table 1 below): the *L*_1_ distance between the vector of true prevalences at the schools and the estimated vector of prevalences using the sampling method, and the number of misclassifications into the incorrect WHO prevalence category. Note that pooling with 25 slides classification accuracy close to the accuracy of 50 slides using the individualed method (only five more misclassifications), whereas dropping to 25 *individualed* results in 22 more misclassifications. Furthermore, the *L*_1_ distance from truth for 25 pooled is somewhere between the *L*_1_ accuracy of the 25 and 50 individualed slides methods, but closer to the 50 individualed accuracy.

We see that the relative benefits of pooling in terms of classification change with the priors and assumptions about varying sensitivity with prevalence (see Reference 6).

## Discussion

Previously, we have shown (in Asymptotic mse ratio of the estimators section) that if one assumes constant and known sensitivities (per smear *sens_1_* and per slide, *sens_4_*) the mse of the pooled estimator is then roughly half that of the individualed estimator at prevalences below roughly 30%. The pooled estimator has a lower mse than the individualed estimator up to roughly 50% prevalence (and higher as the sensitivities decrease, because that lowers the probability of all positive pools). This is of interest because we can read half as many slides to achieve the same accuracy of prevalence estimation in regions with below 30% prevalence. (It may be most important to monitor the prevalence in these regions below 30%, because above 30% it will be easy to determine that treatment of the region is necessary and that appropriate actions be taken.) We see that as the relative difference between the sensitivity from a single smear *sens_1_* and the sensitivity of an entire slide *sens_4_* becomes smaller, the relative benefits of pooling are increased. The DeVlas model can provide insight into when these sensitivities are closer or farther apart.

Furthermore, the DeVlas model allows us to simulate the reality that sensitivity is not constant and known. We see in Simulations section that the pooled estimator generally outperforms the individualed, when using the same number of slides. Varying each parameter value in the DeVlas model allows us to see how the variance, bias, and mse change for the pooled and individualed estimators. The difficulty with estimating prevalence with an unknown sensitivity that changes with prevalence does create a problem for either the pooled or individualed estimators and more work needs to be done to understand how to circumvent this issue.

Latent class (LC) analysis has been used to deal with the issue of unknown test sensitivity. It can be used when several diagnostic tests are available, as was done in Reference 20. Such methods have been extended to the case where sensitivity varies by group (such as location, gender, or age).^21^ We model sensitivity explicitly as a function of prevalence, which may be possible to incorporate into the LC methods.

When using the same number of slides read, for prevalences below, we achieve better accuracy with pooled estimators than the standard individualed estimator for the prevalence of schistosomiasis, even if the test sensitivity does decrease with pooling. The relative benefit of pooling depends upon prevalence, how much the single smear sensitivity (*sens*_1_) differs from the slide sensitivity (*sens*_4_). Further investigation is needed to determine in which regions these benefits are greatest, and whether there is added complexity and cost introduced by the laboratory procedure of creating a pooled slide rather than an individualed slide.

## Figures and Tables

**Table 1:** Comparison of methods using real data

Method	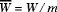	# Misclassified
12 slides, individualed	53	96
25 slides, individualed	37	67
50 slides, individualed	26	45
12 slides, pooled	41	72
25 slides, pooled	30	50

## Appendix: *c*-smear sensitvity

We now compute the sensitivity of the test when taking *c* smears from the same individual, which we call the *c*-smear sensitivity, or *sens_c_*. We use the distributions defined previously in The model section from the DeVlas model. Recall that *h*_0_ is the number of eggs per smear per worm-pair.

where *h* = *ch*_0_, the number of eggs per sample (i.e. slide) per worm−pair

where Γ(.) represents the gamma function, a component of various probability distributions and defined as  for any *z* > 0. The *I*(*n* ≠ 2*x*) denotes the indicator function that equals 1 if (*n* ≠ 2*x*), and 0 otherwise.
